# High intraspecific diversity of *Restorer‐of‐fertility‐like* genes in barley

**DOI:** 10.1111/tpj.14115

**Published:** 2018-11-09

**Authors:** Joanna Melonek, Ruonan Zhou, Philipp E. Bayer, David Edwards, Nils Stein, Ian Small

**Affiliations:** ^1^ ARC Centre of Excellence in Plant Energy Biology School of Molecular Sciences The University of Western Australia Crawley WA Australia; ^2^ Leibniz Institute of Plant Genetics and Crop Plant Research (IPK) Seeland Germany; ^3^ School of Biological Sciences The University of Western Australia Crawley WA Australia; ^4^ School of Agriculture and Environment University of Western Australia Crawley WA Australia

**Keywords:** *Restorer‐of‐fertility‐like* gene, mitochondria, pentatricopeptide repeat protein, cytoplasmic male sterility, hybrid breeding, *Hordeum vulgare*

## Abstract

Nuclear *restorer of fertility* (*Rf*) genes suppress the effects of mitochondrial genes causing cytoplasmic male sterility (CMS), a condition in which plants fail to produce viable pollen. *Rf* genes, many of which encode RNA‐binding pentatricopeptide repeat (PPR) proteins, are applied in hybrid breeding to overcome CMS used to block self‐pollination of the seed parent. Here, we characterise the repertoire of restorer‐of‐fertility‐like (RFL) PPR genes in barley (*Hordeum vulgare*). We found 26 RFL genes in the reference genome (‘Morex’) and an additional 51 putative orthogroups (POGs) in a re‐sequencing data set from 262 barley genotypes and landraces. Whereas the sequences of some POGs are highly conserved across hundreds of barley accessions, the sequences of others are much more variable. High sequence variation strongly correlates with genomic location – the most variable genes are found in a cluster on chromosome 1H. A much higher likelihood of diversifying selection was found for genes within this cluster than for genes present as singlets. This work includes a comprehensive analysis of the patterns of intraspecific variation of RFL genes. The RFL sequences characterised in this study will be useful for the development of new markers for fertility restoration loci.

## Significance statement

*Restorer of fertility* genes are applied in plant breeding to overcome mitochondrially conditioned male sterility in hybrid crop production. Although these genes are known to vary between related species, intraspecific variation in the gene family has not been comprehensively studied. By systematic analysis of restorer of fertility‐like sequences across hundreds of barley accessions, we show that a huge diversity of these RNA‐binding proteins exists within a single species, with strong diversifying selection on the residues known to determine binding specificity.

## **INTRODUCTION**

After wheat, maize and rice, barley is the fourth most important cereal crop in regard to production area, with a world output of 144 million tonnes in 2014 (FAOSTAT). As an abiotic‐stress‐resilient cereal, the seed yield of barley is more stable against seasonal variation than that of wheat and most other small grains. The possibilities of exploiting hybrid heterosis in barley have been explored for decades with the major goals of further increasing crop yield and stability, particularly in marginal environments. The potential heterosis of F1 hybrid varieties in barley has been estimated at about 10% yield gain compared with inbred parental lines (Longin *et al*., 2012; Muhleisen *et al*., 2013).

Three methods have been applied to block self‐pollination of crop plants in hybrid breeding. Manual emasculation of flowers is widely used in hybrid production in maize but, due to flower architecture, this method is not applicable on a commercial scale in barley or wheat. The efficiency of treatment with gametocidal chemicals is strongly influenced by weather conditions and it can also negatively impact yield. In comparison, the use of cytoplasmic male sterility (CMS) can be, once established in a given crop, a labour and cost‐effective way of large scale emasculation of the seed parents of hybrids (Chase, 2007; Chen and Liu, 2014). CMS is induced by mitochondrial genes, the effects of which can be overcome by nuclear restorer of fertility genes (Schnable and Wise, 1998; Hanson and Bentolila, 2004). The majority of known restorer genes belong to the family of pentatricopeptide repeat (PPR) proteins (Dahan and Mireau, 2013; Chen and Liu, 2014; Hu *et al*., 2014; Gaborieau *et al*., 2016). PPR proteins are targeted to mitochondria or chloroplasts where they participate in a plethora of RNA‐associated processes (Barkan and Small, 2014). The PPR family has expanded significantly in plants and, on average, ~500 PPR genes are present in diploid plant genomes (O'Toole *et al*., 2008; Fujii *et al*., 2011; Cheng *et al*., 2016; Sykes *et al*., 2016). Members of the PPR family are identified by the presence of tandem repeats of degenerate 31–36 amino acids, and can be divided into P‐ and PLS‐classes based on the PPR motif structure (Lurin *et al*., 2004; Cheng *et al*., 2016). The P subfamily consists of PPR proteins with canonical 35‐amino‐acid‐long PPR motifs, while the PLS subfamily includes proteins with additional S (for short) and L (for long) motif variants arranged into PLS triplets (Lurin *et al*., 2004; Cheng *et al*., 2016). P‐class PPRs are involved in RNA stabilisation and processing, including 5’ and 3’ RNA cleavage and intron splicing; they are also involved in the initiation of mRNA translation (Meierhoff *et al*., 2003; Raynaud *et al*., 2007; de Longevialle *et al*., 2008; Pfalz *et al*., 2009; Prikryl *et al*., 2011). In comparison, the main function assigned to PLS‐class PPRs is the C‐U editing of organellar transcripts (Okuda *et al*., 2007; Sosso *et al*., 2012; Chateigner‐Boutin *et al*., 2013). PPR proteins recognise their organellar RNA targets in a sequence‐specific manner with base specificity relying on specific amino acids in each PPR motif, with the strongest effect observed for residues at positions 5 and 35 (Fujii *et al*., 2011; Barkan *et al*., 2012; Takenaka *et al*., 2013; Yagi *et al*., 2013; Miranda *et al*., 2018).

Rf proteins are generally P‐class PPR proteins (Dahan and Mireau, 2013; Gaborieau *et al*., 2016) and, although plant genomes encode hundreds of P‐class PPR proteins, on average, only ~10% of them belong to the restorer‐of‐fertility‐like (RFL) clade (Fujii *et al*., 2011; Melonek *et al*., 2016; Sykes *et al*., 2016). Characteristic features that discriminate RFL genes from other P‐class PPR proteins include clustering at a small number of genomic locations, and their relatively high number of PPR motifs with close similarity to the PPR consensus (Fujii *et al*., 2011; Melonek *et al*., 2016). The most remarkable attribute of RFL proteins is their evolutionary plasticity, reflected in much higher evolution rates compared with any other group of PPR proteins, with diversifying selection acting particularly on amino acid residues involved in binding to RNA targets (Fujii *et al*., 2011). The role of RFL proteins in suppressing CMS by blocking expression of CMS‐associated ORFs has been proposed as a possible explanation for their diversity and unusual evolutionary behaviour (Chase, 2007). The co‐evolution of nuclear RFL genes with CMS‐inducing mitochondrial genes has been compared with the co‐evolution of pathogen effectors and resistance (*R*) genes in plant–pathogen interactions, described as an ‘evolutionary arms race’ between the mitochondrial and nuclear genomes (Touzet and Budar, 2004; Dahan and Mireau, 2013).

The development of a CMS‐based hybrid breeding system requires three types of breeding lines: a ‘female line’ or cytoplasmic male sterile line, which carries a mitochondrial gene that causes CMS, a maintainer line that is required for propagating the sterile line and a restorer line, which carries a nuclear *Rf* gene that restores male fertility by suppressing the action of the CMS‐causing gene. Two CMS cytoplasms, designated *msm1* and *msm2*, respectively, were found as natural variants of the wild progenitor of barley *Hordeum vulgare* ssp. *spontaneum* (C. Koch) Thellung (Ahokas, 1979, 1982). So far, only a single dominant restorer gene *Rfm1* (*restorer of fertility in msm1*) has been identified that restores the fertility of both *msm1* and *msm2* cytoplasm (Ahokas, 1980a,b, 1982), and the *Rfm1*‐CMS system has been used to develop the HYVIDO^®^ family of high‐yielding 6‐row winter barley by Syngenta (Rizzolatti *et al*., 2017). As these hybrids were shown to display several advantages over non‐hybrid varieties, including consistency in the seed yield from year to year by better overcoming severe weather conditions and higher resistance to diseases, the prospective benefits of breeding hybrid varieties in barley based on CMS are promising (Muhleisen *et al*., 2013, 2014a,b). However, the application of *Rfm1*‐CMS in hybrid breeding in barley is limited due to its thermosensitivity, as spontaneous fertility restoration in the absence of the *Rfm1* gene occurs during periods of higher temperatures around heading and flowering time (Bernhard *et al*., 2017). Therefore, the identification of new sources of CMS and alternative restorer genes, the most likely origin of which will be RFL genes from wild barley relatives, will be crucial for the development of new hybrid varieties.

In this study, the RFL family in the genus *Hordeum* was comprehensively characterised, and the conservation and sequence variation of identified RFLs across hundreds of barley accessions, landraces and wild relatives analysed.

## **RESULTS**

### Identification of RFL genes in the genus *Hordeum*

**Figure 1:**
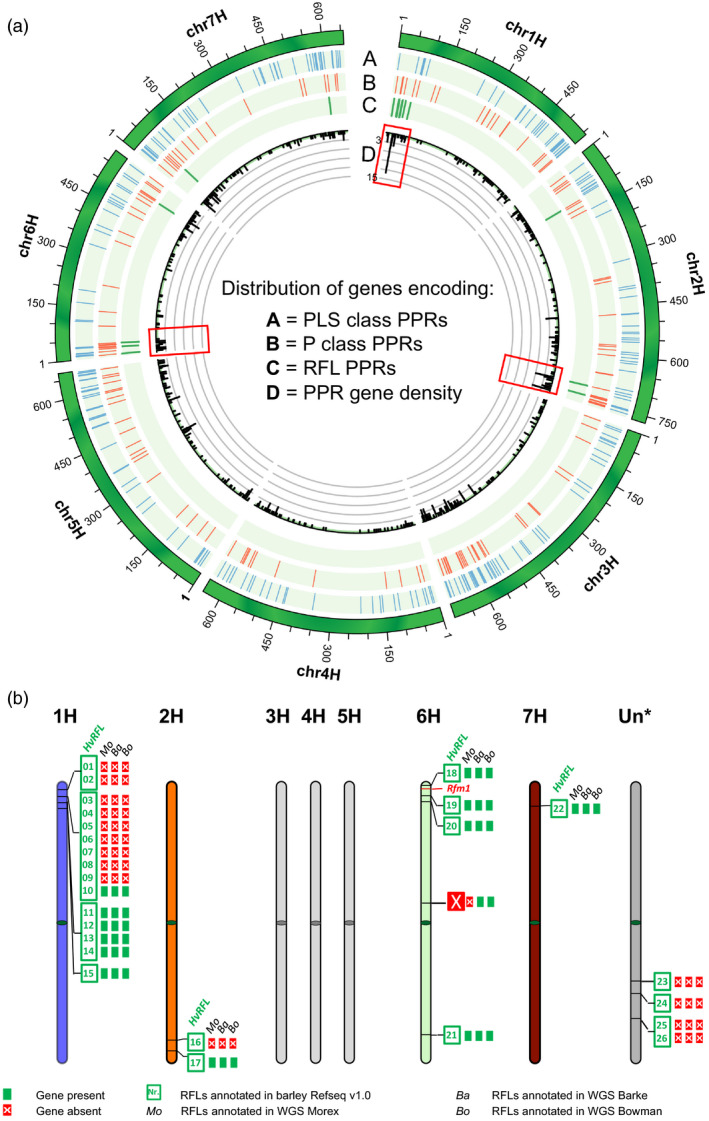
Genome‐wide distribution of the pentatricopeptide repeat (PPR) and restorer‐of‐fertility‐like (RFL) family members in the *Hordeum vulgare* cv. ‘Morex’ Refseqv1.0 reference genome. (a) Circos diagram illustrating the genome‐wide distribution of the PLS‐ and P‐class PPRs as well as RFL sequences in barley. The density of PPR genes per 2‐Mb windows along the chromosomes is shown. Red frames indicate the genomic regions showing unusually high RFL gene density.(b) Schematic drawing showing the locations of the identified 26 RFLs in the newest version of the *H. vulgare* cv. ‘Morex’ Refseqv1.0 genome (Mascher *et al*., 2017) as well as whole‐genome shotgun (WGS) data sets of cvs. ‘Morex’ (Mo), ‘Barke’ (Ba) and ‘Bowman’ (Bo) (The International Barley Genome Sequencing Consortium, 2012). The genomic location of the mapped position of the *Rfm1* locus (Ui *et al*., 2015; Rizzolatti *et al*., 2017) is shown on the short arm of chromosome 6H.

For *H. vulgare* cv. ‘Morex’ the newest barley reference genome Refseqv1.0 (Mascher *et al*., 2017) was used. For comparison, previously published draft whole‐genome shotgun (WGS) assemblies of *H. vulgare* cvs. ‘Morex’, ‘Barke’ and ‘Bowman’ (The International Barley Genome Sequencing Consortium, 2012) were included in the study (Table 1). The genomic sequences were analysed to identify RFL sequences as previously described for rice (Melonek *et al*., 2016). In total, 245 PLS‐class and 215 P‐class PPR genes were identified in the *H. vulgare* cv. ‘Morex’ reference genome (Table 1). These genes were found to be distributed across all seven barley chromosomes (Figure 1a). Analysis of PPR gene density across the genome revealed the presence of two PPR‐rich regions on chromosomes 1H and 2H showing higher gene density compared with other regions in the barley genome (Figure 1a).

**Table 1:** Identified RFL sequences in barley ‘Morex’ Refseqv1.0 reference genome and WGS data sets of ‘Morex’, ‘Barke’ and ‘Bowman’

*#*	Species	Genomes coded names	Data set type	References	Identification of ORFs containing PPR motifs				# of RFLs (10 or more PPR motifs)
					# ORFs/6 frame translations (× 1000)	# ORFs with PPR repeats	# ORFs with PLS‐class PPR repeats (> 240)^a^	# ORFs with P‐class PPR repeats (> 100)^a^	
1.	*Hordeum vulgare cv*. ‘Morex’	HvMo	WGA	(Mascher *et al*., 2017)	10 908	953	245	215	26
2.	*Hordeum vulgare cv*. ‘Morex’	HvM	WGS	(The International Barley Genome Sequencing Consortium, 2012)	2824	683	222	116	12
3.	*Hordeum vulgare cv*. ‘Bowman’	HvBo	WGS	(The International Barley Genome Sequencing Consortium, 2012)	2988	659	209	115	13
4.	*Hordeum vulgare cv*. ‘Barke’	HvBa	WGS	(The International Barley Genome Sequencing Consortium, 2012)	2613	633	160	94	13

a PPR protein scores as judged from *hmmsearch*.

PPR, pentatricopeptide repeat; RFL, restorer‐of‐fertility‐like.

RFL sequences were identified by two approaches: phylogenetic analysis; and by inferring orthologous groups with OrthoMCL‐DB (Chen *et al*., 2006; Table S1). In total, 55 RFL sequences with amino acid length ranging from 925 to 93 were identified (Table S1). Of these, 26 represent genes encoding putative full‐length PPR proteins with 10 or more PPR motifs (Tables 1 and S1).

The analysis of the WGS draft assemblies of *H. vulgare* cvs. ‘Morex’, ‘Barke’ and ‘Bowman’ identified 12 RFL genes in ‘Morex’ and 13 in the two latter accessions (Figure S1; Tables 1 and S2). The majority of the missing RFL sequences correspond to genes in the cluster on chromosome 1H in ‘Morex’ (Table 2). Detailed sequence analyses revealed that seven RFL genes were identical between all three cultivars (sequence identity > 99%), three genes were identical between ‘Bowman’ and ‘Barke’ (sequence identity 100%), and one gene was identical between ‘Bowman’ and ‘Morex’ (sequence identity 100%; Table S2). One gene, HvBo_RFL11 = HvBa_RFL11, was found only in the cvs. ‘Barke’ and ‘Bowman’ (Table S2). In regard to ‘Morex’, for all 12 RFL genes identified in the WGS assembly, a corresponding gene was found in the barley Refseqv1.0 reference genome (Figures 1 and S1; Table S2).

**Table 2:** Overview of RFL sequences identified in the ‘Morex’ Refseqv1.0 reference genome and WGS data sets of ‘Morex’, ‘Barke’ and ‘Bowman’

Data set	Chromosome H1															2H		6H				Unknown					# of extra genes	Total
	Sub‐cluster 1		Sub‐cluster 2								Sub‐cluster 3																	
	HvRFL01	HvRFL02	HvRFL03	HvRFL04	HvRFL05	HvRFL06	HvRFL07	HvRFL08	HvRFL09	HvRFL10	HvRFL11	HvRFL12	HvRFL13	HvRFL14	HvRFL15	HvRFL16	HvRFL17	HvRFL18	HvRFL19	HvRFL20	HvRFL21	HvRFL22	HvRFL23	HvRFL24	HvRFL25	HvRFL26		
‘Morex’ Refseqv1.0	1	1	1	1	1	1	1	1	1	1	1	1	1	1	1	1	1	1	1	1	1	1	1	1	1	1	0	26
WGS ‘Morex’	0	0	0	0	0	0	0	0	0	1	1	1	1	1	1	1	0	1	1	1	1	1	0	0	0	0	0	12
WGS ‘Barke’	0	0	0	0	0	0	0	0	0	1	1	1	1	1	1	1	0	1	1	1	1	1	0	0	0	0	1	13
WGS ‘Bowman’	0	0	0	0	0	0	0	0	0	1	1	1	1	1	1	1	0	1	1	1	1	1	0	0	0	0	1	13

### Genome‐wide distribution of RFLs in *Hordeum vulgare* cv. ‘Morex’

Twenty‐two of the identified ‘Morex’ RFLs were located on four chromosomes (Figure 1b; Table 3), and four sequences were located on unanchored scaffolds (chrUn; Figure 1b; Table 3). The highest number of RFLs was present on chromosome 1H, where 13 RFLs were organised into three sub‐clusters and one gene (*HvRFL15)* was found as a singlet (Figure 1b; Table 3). The biggest sub‐cluster on chromosome 1H (sub‐cluster 2) spanned a region of ~2 Mbp and contained eight full‐length RFL sequences (Figure 1b; Table 3). Sequence clustering with CD‐HIT suggested that all four RFLs (RFL23–26) located on unanchored scaffolds most likely originate from chromosome 1H (Table S3). Including these genes, the total number of RFL genes in 1H sub‐cluster 2 may be as high as 12 (Table 3). Sub‐cluster 1 and sub‐cluster 3 on chromosome 1H were composed of two and four RFLs, respectively (Figure 1; Table 3). All four RFL genes located on chromosome 6H were separated by long DNA stretches ranging from 10 to 23 Mbp, and thus were classified as singlets (Figure 1b; Table 3). Sequence alignment of the 26 RFL sequences revealed that one of the genes identified on chromosome 6H (*HvRFL21*) shared 70% sequence identity to *HvRFL14* and *HvRFL11*, both located on chromosome 1H, suggesting a possible misassembly of this gene on chromosome 6H or a recent chromosomal relocation of the gene within the barley genome (Figure 1b). A single RFL gene (*HvRFL22*) was found on chromosome 7H (Figure 1b). The genomic distances between the RFL genes located in the gene clusters on chromosome 1H and 2H were much shorter compared with the distances between RFLs present as singlets or any other type of PPR genes located anywhere else in the genome as visualised by the density plot (Figure 1a). The mapped *Rfm1* interval (Ui *et al*., 2015) is indicated on the short arm of chromosome 6H (Figure 1b) and does not include any of the RFL sequences.

**Table 3:** The repertoire of RFL proteins in *Hordeum vulgare* cv. ‘Morex’ Refseqv1.0 genome

#	RFL ID	Gene name	Genomic location	Cluster/sensing	Physical position [start–end] (orientation)	Gene length (bp)	PPR motif structure	mTP yes [length amino acids)	Possible pseudogene
1.	HvRFL01	HORVU1Hr1G004750	chr1H	Sub‐cluster 1	[9851312–9848892] (REVERSE)	2421	56‐P‐46‐P‐P‐P‐P‐P‐P‐P‐P‐P‐P‐P‐P‐P‐P‐P‐P‐P‐P‐38	Yes [111]	
2.	HvRFL02	Not annotated	chr1H		[9907009–9904493] (REVERSE)	2517	56‐P‐46‐P‐P‐P‐P‐P‐P‐P‐P‐P‐P‐P‐315	Yes [25]	Yes
3.	HvRFL03	HORVU1Hr1G010890	chr1H	Sub‐cluster 2	[25542037–25539329] (REVERSE)	2709	199‐P‐P‐P‐P‐P‐P‐P‐P‐P‐P‐P‐P‐P‐P‐P‐P‐P‐P‐70	Yes [18]	
4.	HvRFL04	HORVU1Hr1G010970	chr1H		[25974315–25976849] (FORWARD)	2535	58‐P‐48‐P‐P‐P‐P‐P‐P‐P‐P‐P‐P‐P‐P‐P‐P‐P‐P‐P‐P‐70	Yes [21]	
5.	HvRFL05	HORVU1Hr1G011020	chr1H		[26214086–26211741] (REVERSE)	2346	149‐P‐P‐P‐P‐P‐P‐P‐P‐P‐P‐P‐P‐P‐P‐P‐P‐70	Yes [53]	
6.	HvRFL06	Not annotated	chr1H		[26421244–26418548] (REVERSE)	2697	195‐P‐P‐P‐P‐P‐P‐P‐P‐P‐P‐P‐P‐P‐P‐P‐P‐P‐P‐P‐35	Yes [77]	
7.	HvRFL07	Not annotated	chr1H		[26485385–26488081] (FORWARD)	2697	195‐P‐P‐P‐P‐P‐P‐P‐P‐P‐P‐P‐P‐P‐P‐P‐P‐P‐P‐P‐35	Yes [77]	
8.	HvRFL08	HORVU1Hr1G011160	chr1H		[26659630–26662332] (FORWARD)	2703	197‐P‐P‐P‐P‐P‐P‐P‐P‐P‐P‐P‐P‐P‐P‐P‐P‐P‐P‐70	Yes [18]	
9.	HvRFL09	HORVU1Hr1G011300	chr1H		[27069847–27072621] (FORWARD)	2775	221‐P‐P‐P‐P‐P‐P‐P‐P‐P‐P‐P‐P‐P‐P‐P‐P‐P‐P‐70	Yes [18]	
10.	HvRFL10	HORVU1Hr1G011400	chr1H		[27458351–27456489] (REVERSE)	1863	68‐P‐48‐P‐P‐P‐P‐P‐P‐P‐P‐P‐P‐P‐35‐P‐8	Yes [107]	
11.	HvRFL11	HORVU1Hr1G013810	chr1H	Sub‐cluster 3	[37317192–37319720] (FORWARD)	2529	140‐P‐P‐P‐P‐P‐P‐P‐P‐P‐P‐P‐P‐P‐P‐P‐P‐P‐P‐70	Yes [32]	
12.	HvRFL12	HORVU1Hr1G014030	chr1H		[38711706–38709145] (REVERSE)	2562	150‐P‐P‐P‐P‐P‐P‐P‐P‐P‐P‐P‐P‐P‐P‐P‐P‐P‐P‐70	Yes [19]	
13.	HvRFL13	HORVU1Hr1G014040	chr1H		[38766653–38769199] (FORWARD)	2547	145‐P‐P‐P‐P‐P‐P‐P‐P‐P‐P‐P‐P‐P‐P‐P‐P‐P‐P‐35‐P	Yes [46]	
14.	HvRFL14	HORVU1Hr1G014060	chr1H		[38811031–38813742] (FORWARD)	2712	198‐P‐P‐P‐P‐P‐P‐P‐P‐P‐P‐P‐P‐P‐P‐P‐P‐P‐P‐70	Yes [33]	
15.	HvRFL15	Not annotated	chr1H	Singlet	[48954576–48956174] (FORWARD)	1599	5‐P‐P‐P‐P‐P‐P‐P‐P‐P‐P‐P‐P‐P‐P‐37	No	Yes
16.	HvRFL16	HORVU2Hr1G105460	chr2H	Cluster 1	[706791942–706790239] (REVERSE)	1704	44‐P‐P‐P‐P‐P‐P‐P‐53‐P‐P‐P‐P‐P‐50	No	Yes
17.	HvRFL17	HORVU2Hr1G116650	chr2H		[738768328–738766658] (REVERSE)	1671	P‐P‐P‐P‐P‐P‐P‐P‐P‐P‐P‐P‐P‐P‐86	No	Yes
18.	HvRFL18	HORVU6Hr1G002890	chr6H	Singlet	[7033847–7031583] (REVERSE)	2265	124‐P‐P‐P‐P‐P‐P‐P‐P‐P‐P‐P‐P‐31‐P‐P‐P‐P‐36	Yes [25]	
19.	HvRFL19	HORVU6Hr1G014310	chr6H	Singlet	[30413113–30415251] (FORWARD)	2139	103‐P‐P‐P‐P‐P‐P‐P‐P‐P‐P‐P‐P‐P‐P‐P‐P‐P‐11	Yes [46]	Yes
20.	HvRFL20	HORVU6Hr1G014310	chr6H	Singlet	[43353403–43354881] (FORWARD)	1479	101‐P‐P‐P‐P‐P‐P‐P‐P‐P‐P‐P‐6	No	Yes
21.	HvRFL21	HORVU6Hr1G076780	chr6H	Singlet	[527185061–527182566] (REVERSE)	2496	128‐P‐P‐P‐P‐P‐P‐P‐P‐P‐P‐P‐P‐P‐P‐P‐P‐P‐P‐P‐35	Yes [10]	
22.	HvRFL22	HORVU7Hr1G029840	chr7H	Singlet	[56755710–56753593] (REVERSE)	2118	36‐P‐P‐P‐P‐P‐P‐P‐P‐P‐P‐P‐P‐P‐P‐P‐P‐P‐P‐38	No	Yes
23.	HvRFL23	Not annotated	Unknown^a^	Unknown	[177465399–177467981] (FORWARD)	2583	157‐P‐P‐P‐P‐P‐P‐P‐P‐P‐P‐P‐P‐P‐P‐P‐P‐P‐P‐70	Yes [35]	
24.	HvRFL24	HORVU0Hr1G031920	Unknown^a^	Unknown	[190422976–190420268] (REVERSE)	2709	199‐P‐P‐P‐P‐P‐P‐P‐P‐P‐P‐P‐P‐P‐P‐P‐P‐P‐P‐70	Yes [18]	
25.	HvRFL25	Not annotated	Unknown^a^	Unknown	[210337547–210339712] (FORWARD)	2166	126‐P‐P‐P‐P‐P‐P‐P‐P‐P‐P‐P‐P‐P‐P‐P‐P‐P	No	Yes
26.	HvRFL26	HORVU0Hr1G035310	Unknown^a^	Unknown	[210357231–210359657] (FORWARD)	2427	105‐P‐P‐P‐P‐P‐P‐P‐P‐P‐P‐P‐P‐P‐P‐P‐P‐P‐P‐70	Yes [35]	

Genes most likely located in RFL sub‐cluster 2 on chromosome 1H (based on CD‐HIT clustering).

mTP, mitochondrial targeting sequence; PPR, pentatricopeptide repeat; RFL, restorer‐of‐fertility‐like.

### Characterisation of identified RFL sequences: functional genes versus pseudogenes

The 26 identified HvRFL sequences contain between 11 and 19 PPR motifs (Table 3). For 16 of them a mitochondrial localisation (Small *et al*., 2004) was predicted using *Predotar* (Table 3), in agreement with the expected mitochondrial location of RFL proteins. Nine of the RFL sequences may represent pseudogenes as the encoded proteins either lack a predicted mitochondrial targeting sequence (mTP) or are truncated in the middle of a PPR motif (Table 3). In addition to these long RFL sequences encoding proteins composed of 10 or more PPR motifs, the genomic regions carrying RFL genes contain an additional 29 short RFL sequences (Table S1). Such partial RFL sequences may represent remnants of RFL genes disrupted by unequal crossing‐over reported to occur frequently during recombination events within RFL clusters (Melonek *et al*., 2016).

### RFL gene variation across 262 barley accessions

**Figure 2:**
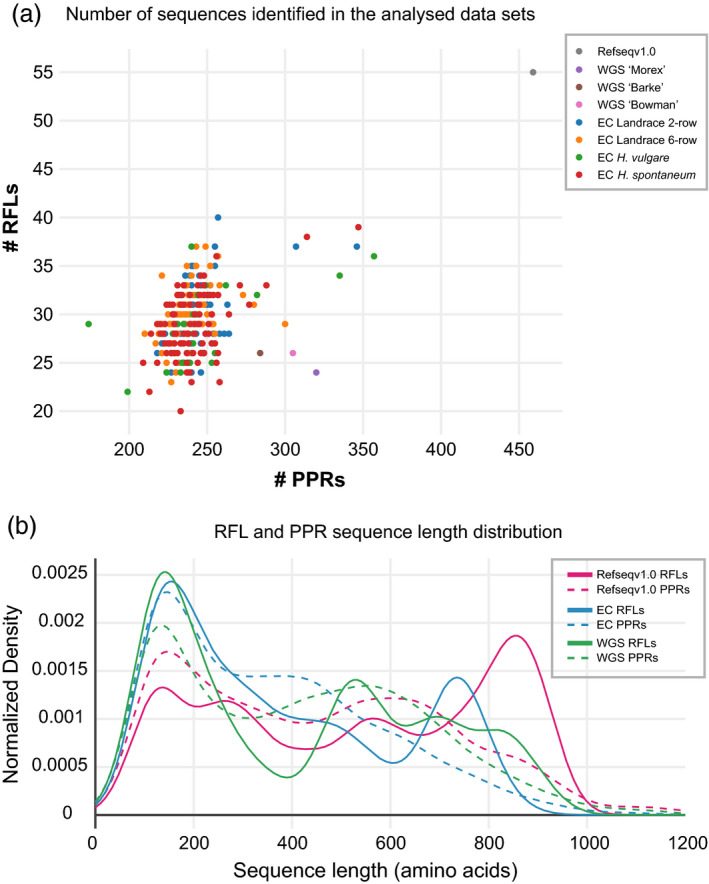
Characterisation of restorer‐of‐fertility‐like (RFL) sequences recovered from various barley sequence data sets.(a) Scatter plot illustrating the number of identified RFLs in each barley accession compared with the total number of pentatricopeptide repeat (PPR) sequences. The plot shows results from the analysis of Refseqv1.0 genome as well as from whole‐genome shotgun (WGS) assemblies and exome capture (EC) data sets.(b) Length distribution of PPR and RFL‐type ORFs in the Refseqv1.0 genome, EC data sets and WGS data sets. Gaussian kernel density estimates of the ORF length distributions were generated with the *sklearn.neighbors.KernelDensity* estimator version 0.18.1 (Pedregosa *et al*., 2011), and the data were visualised in an offline version of *plotly* (https://plot.ly/). PPR ORFs longer than 279 nucleotides (= 93 amino acids) were included in the analysis.

Target enrichment re‐sequencing data from 262 barley accessions (Mascher *et al*., 2013; Russell *et al*., 2016) were analysed to estimate the sequence variation of RFL genes in the genus *Hordeum*. One‐hundred and sixty‐eight data sets were from barley cultivars and landraces, and 94 were from wild *Hordeum spontaneum* accessions (Table S4). *De novo* assembly of captured reads was performed with MaSuRCA (Zimin *et al*., 2013), and the obtained sequence scaffolds were screened for the presence of PPR ORFs of P‐ and PLS‐type, the number of which varied from 174 to 357 and 186 to 383, respectively (Table S5). About 20–40 RFL ORFs were found in the analysed accessions (Table S5) compared with the 55 RFL ORFs found in the ‘Morex’ Refseqv1.0 genome (Figure 2a), suggesting that only about half of the RFL sequences were captured and assembled. The captured RFL ORFs are, on average, shorter, as illustrated in Figure 2(b). Whereas the length distributions for PPR ORFs are relatively consistent between the different data sets, the exome capture (EC) and genome shotgun assemblies contain a dearth of long RFL sequences (over 800 amino acids) and a large excess of short RFL sequences (less than 200 amino acids).

**Figure 3:**
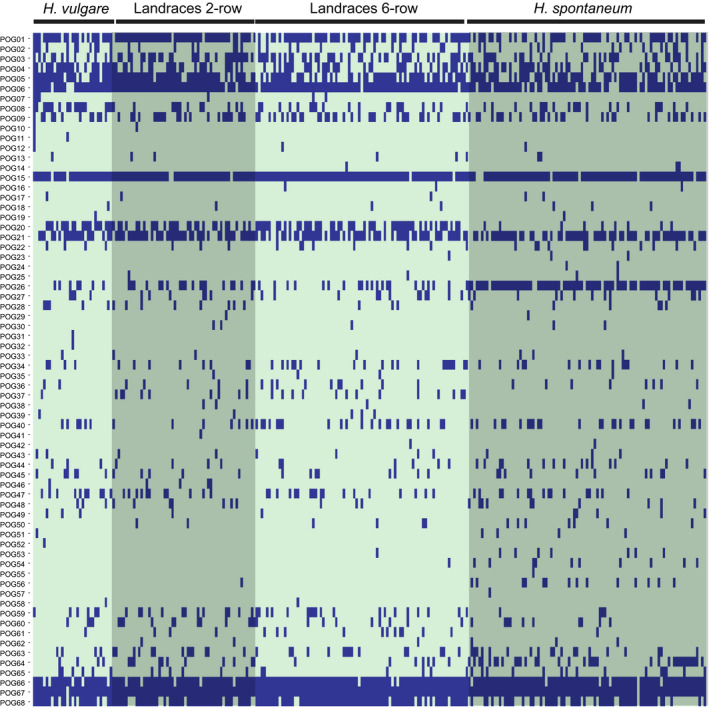
Identifying the repertoire of putative orthogroups (POGs) across 262 barley accessions.Matrix illustrating the presence (blue bar) of representative sequences of 68 reference POG sequences identified by hierarchical cluster analysis (HCA) of 7704 sequences identified in data sets from 262 barley accessions. The matrix was visualised in an offline version of *plotly* (https://plot.ly/).

This observed sequence fragmentation could be due to the repetitive nature of highly similar RFL sequences, which creates ambiguities in alignment and computational challenges during assembly (Table S6 lists examples of partial exon assemblies). These issues make it impossible to determine whether a sequence is truly absent in a given accession or whether it has simply escaped detection by the current approach. With that caveat in mind, a hierarchical cluster analysis (HCA) with CD‐HIT (Huang *et al*., 2010) was performed to assess gene conservation and look for new RFL variants within the 262 barley accessions. During this process, similar RFL sequences were iteratively grouped into clusters representing putative orthogroups (POGs). Ideally, we were aiming to assign sequences of a single RFL gene into one POG across all 262 accessions but, due to extremely high sequence similarity between some RFL genes, it is unavoidable that in some cases a POG will contain sequences from several distinct genes. For example, POG3 contains both HvRFL06 and HvRFL07 (Table S7) as the two sequences are identical. By using the described approach across all 262 EC data sets and the ‘Morex’ Refseqv1.0, 68 putative POGs were identified (Figure 3;Table S7). Fifteen POGs each correspond to a single gene found in the Refseqv1.0 genome, two contain multiple ‘Morex’ reference genes and 51 POGs contain no Refseqv1.0 genes (Table S7). Four POGs (POG67, POG15, POG66 and POG06) were found in more than 90% of the accessions and the WGS assemblies of ‘Barke’ and ‘Morex’ (Figure 3; Table S8). These correspond to ‘Morex’ HvRFL18, HvRFL19, HvRFL21 and HvRFL12, respectively (Table S8), which, apart from HvRFL12, were annotated on chromosome 6H as singlets (Table 3). Sequence alignments of these genes revealed high sequence conservation with very few amino acid substitutions (Figure S2a). In comparison, three genes located on chromosome 1H in ‘Morex’ POG68, POG05 and POG01 corresponding to HvRFL11, HvRFL09 and HvRFL01, respectively, were found in 70% of the accessions (Table S8) and show high sequence divergence (Figure S2b). The remaining 61 POGs were found in 50% or less of the accessions, and show different levels of sequence variability (Table S8).

### Estimation of dN/dS rate ratio of barley RFL proteins to assess positive selection

**Figure 4:**
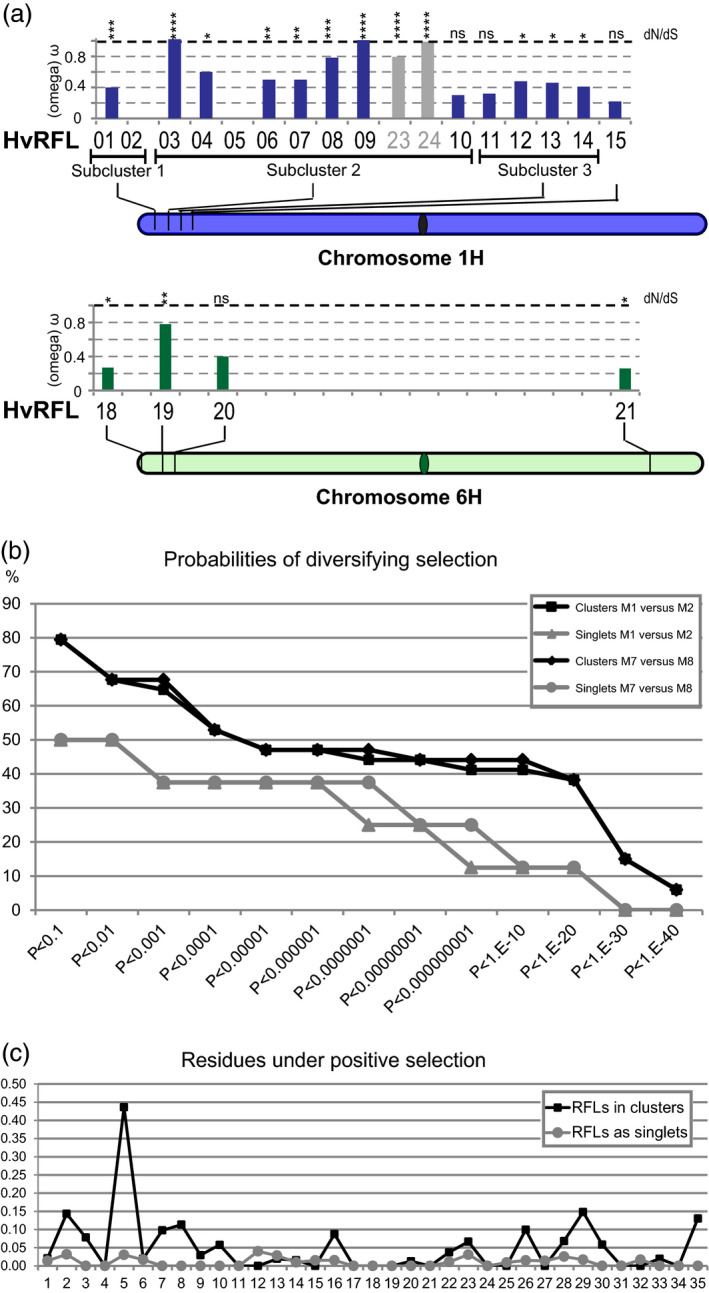
Diversifying selection acting on barley restorer‐of‐fertility‐like (RFL) genes.(a) Average ω values (Model 0) calculated for each HvRFL protein and plotted along the chromosomal positions of corresponding genes along with probabilities of diversifying selection calculated with CODEML. HvRFL23 and HvRFL24 are depicted within sub‐cluster 2 on chromosome 1H based on CD‐HIT clustering, but their position within the sub‐cluster is arbitrary. *P < 0.1, **P < 1.E‐5, ***P < 1.E‐10, ****P < 1.E‐20, ns – no probabilities of diversifying selection.(b) Cumulative proportions of genes that fit models allowing diversifying selection (M2 or M8) better than models assuming only purifying/neutral evolution (M1 or M7), respectively at different *P*‐values. (c) Bayes Empirical Bayes (BEB) probabilities of positive selection mapped onto positions of a pentatricopeptide repeat (PPR) motif. The line chart displays mean positive‐selection probabilities at each amino acid position within the PPR motif from either RFL genes organised in clusters or present in the genome as singlets.

To measure the strength and mode of natural selection acting on barley RFL genes, the ratio of non‐synonymous (dN) to synonymous (dS) substitutions (ω = dN/dS) was calculated (Table S9). First, we estimated the average ω values for each RFL gene by using the M0 model in the CODEML package (Yang, 2007), which averages the ω ratio across all sites in a protein (Jeffares *et al*., 2015). We observed that the average ω values for RFL genes correlate with their genomic location (Figure 4a). In particular, RFLs located within sub‐cluster 2 on chromosome 1H, which contains the highest number of RFLs among all clusters identified in the barley reference genome, show elevated ω values reaching 1 (Figure 4a; Table S9). The lowest ω values (ω = 0.22) were reported for two singlets, *HvRFL15* and *HvRFL21*, located on chromosomes 1H and 6H, respectively (Figure 4a; Table S9). This indicates that purifying rather than diversifying selection is acting on them (Figure 4a).

The probabilities of diversifying selection (dN/dS ratios) for POGs identified by the HCA approach were calculated and compared with predictions from codon substitution models assuming only purifying/neutral evolution or also allowing diversifying selection. Nearly one‐third of analysed POGs showed very strong indications of positive selection (*P *< 1.E‐11 Table S10). In contrast, for 10 genes no evidence of diversifying selection was found (Table S10). Comparison of POGs located within a cluster or present as singlets revealed that genes located in clusters show much higher probabilities of diversifying selection compared with singlets (Figure 4a and b).

Amino acid residues within PPR motifs of RFL proteins that are under positive selection were predicted (Figure 4c) by using the Bayes Empirical Bayes (BEB) approach (Yang *et al*., 2005) implemented in CODEML. In particular, positions 5 and 35 but also 2, 7, 8 and 29 of each PPR motif were found to be under much stronger diversifying selection compared with other residues (Figure 4c). These probabilities of diversifying selection, acting on PPR residues reported to be involved directly in contact with the target RNA, i.e. positions 5 and 35 (Barkan *et al*., 2012), were much higher for POGs organised in clusters than as singlets (Figure 4c).

### Interspecific RFL sequence conservation and synteny

To study RFL gene conservation in other *Hordeum* species, RFL sequences were identified in draft WGS assemblies of *Hordeum pubiflorum*, also known as Antarctic barley, native to South America, and *Hordeum bulbosum*, another wild relative of cultivated barley. These were compared with RFL sequences found in the ‘Morex’ Refseqv1.0 reference genome. Eight of the nine RFLs found in the *H. bulbosum* genome show 78–93% identity with RFLs from *H. vulgare* ‘Morex’ (Figure S1; Table S11). Out of 22 sequences identified in *H. pubiflorum*, only 10 share 64–100% identity with RFL sequences from ‘Morex’ (Table S11), and the remaining 10 sequences group together and show low sequence similarity to *H. vulgare* RFL sequences (Table S11). This result might reflect the greater genetic distance between *H. vulgare* and *H. pubiflorum* than between *H. vulgare* and *H. bulbosum*.

**Figure 5:**
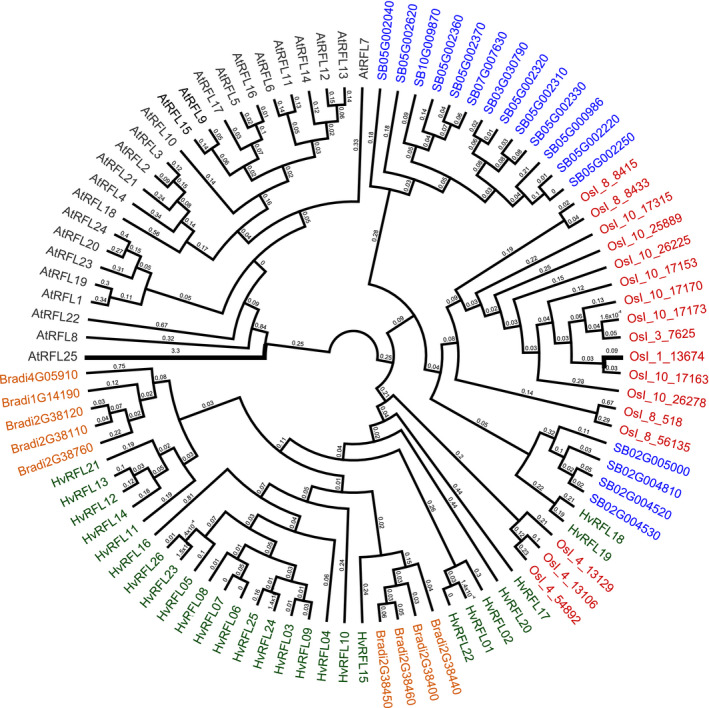
Phylogenetic relationships between restorer‐of‐fertility‐like (RFLs) from *Hordeum vulgare* cv. ‘Morex’ (green), *Sorghum bicolor* (blue), *Oryza sativa* ssp. *indica* (red) and *Brachypodium distachyon* (orange).Arabidopsis RFLs were added as an outgroup (black). Protein sequences were aligned with Muscle v.3.8.31 (Edgar, 2004), and the cladogram was built with FastTree (Price *et al*., 2009) and visualised in *Geneious* (www.geneious.com).

For a broader interspecific comparison, the set of barley Refseqv1.0 reference RFLs was compared with RFLs identified in three other grass species: *Sorghum bicolor* (Fujii *et al*., 2011); *Brachypodium distachyon* (Fujii *et al*., 2011); and *Oryza sativa* ssp. *indica* (Melonek *et al*., 2016; Figure 5). Arabidopsis RFLs were included as an outgroup (Figure 5). Clusters of RFLs from one species are grouped with whole clusters or larger groups of RFL sequences from other species (Figure 5). Within a species, RFL sequences located on the same chromosome show higher sequence similarity to each other than to RFL sequences located on other chromosomes, for example, all sorghum RFL sequences located on chromosome 5 form one group and sequences located on chromosome 2 form another group (Figure 5). Interestingly, *HvRFL18* and *HvRFL19* located on chromosome 6H and identified as highly conserved sequences in barley show high sequence similarity to four sorghum RFLs located on chromosome 2 (Figure 5). Moreover, the majority of RFLs from chromosome 1H cluster in barley are grouped with RFLs located on chromosome 2 in *Brachypodium* (Figure 5). This indicates that these genes might have originated from a common ancestor cluster.

**Figure 6:**
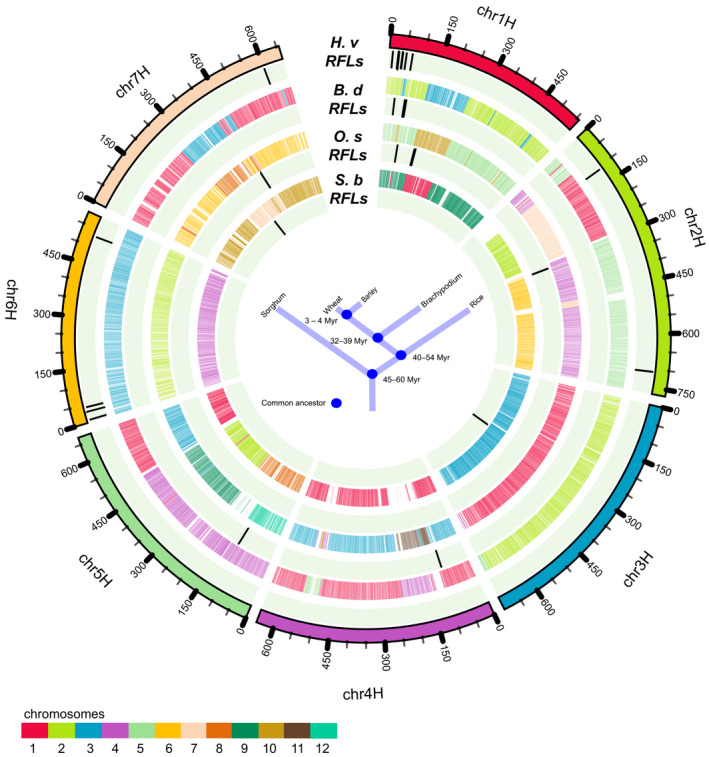
Circos diagram showing synteny between *Hordeum vulgare*,* Brachypodium distachyon*,* Oryza sativa* ssp. *indica* and *Sorghum bicolor* chromosomes as reported earlier (The International Barley Genome Sequencing Consortium, 2012). Genomic locations of restorer‐of‐fertility‐like (RFL) gene clusters are shown as black vertical bars.

In addition to different levels of sequence divergence, differences in level of synteny between RFL clusters were also observed among the analysed species and *H. vulgare* ‘Morex’ (Figure 6). Whereas genomic locations of RFL clusters in *H. vulgare*,* B. distachyon* and *O. sativa* ssp. *indica* partially overlap, no synteny between RFL clusters identified in *H. vulgare* and *S. bicolor* was observed (Figure 6).

## **DISCUSSION**

Over the last few years, as a result of rapid advances in sequencing technologies and computational techniques, increasing numbers of high‐quality plant genome sequences have become available. Among them is the first high‐quality reference (Refseqv1.0) genome of barley (Mascher *et al*., 2017). It is now possible in cereals to characterise large gene families that have long been known and extensively studied in model plants such as Arabidopsis or rice. Some of these gene families are of agronomic importance, and thus the knowledge gains are expected to advance the breeding of new varieties with higher yield and better tolerance to changing environments. One such family is the PPR family, in particular a subclade of it referred to as RFL proteins. *Rf* genes have played a crucial role in the success of hybrid rice varieties (Huang *et al*., 2014). Therefore, the identification of restorer lines carrying strong *Rf* genes is likely to be helpful for further development of commercial CMS‐based restoration systems in barley.

We initially focused on the analysis of the PPR and RFL families in cultivated barley, represented by *H. vulgare* cv. ‘Morex’. The pool of 460 PPR proteins identified in the barley Refseqv1.0 reference genome is similar to the number of PPRs identified in other diploid plant genomes such as Arabidopsis and rice (Cheng *et al*., 2016). The number of identified RFL proteins (26) is also similar to that observed in Arabidopsis and rice (Fujii *et al*., 2011; Melonek *et al*., 2016), but is fewer than the ~30–50 RFL proteins comprised of 10 or more PPR motifs that were found to be encoded by each of the three bread wheat (*Triticum aestivum* Chinese Spring CS42) sub‐genomes (The International Wheat Genome Sequencing Consortium, 2018).

As reported previously for rice and bread wheat, where ~90% of the RFL genes were found in clusters (Melonek *et al*., 2016; The International Wheat Genome Sequencing Consortium, 2018), barley RFL genes are organised in two clusters, with sub‐cluster 2 on chromosome 1H being by far the largest. Within each cluster the genes show a close relationship, such that RFL sequences originating from the same genomic region show higher sequence similarity to each other than to sequences located on other chromosomes. This feature, typical of RFL genes, distinguishes them from other PPR genes that do not show such clustering. Regions of the barley genome carrying RFL clusters show higher PPR gene density than regions with other types of PPR genes. The origin of such RFL‐rich regions can be explained by the proposed mechanism of RFL gene expansion by tandem duplications and unequal crossover (Dahan and Mireau, 2013; Melonek *et al*., 2016), which generate sequence variation that fuels the ‘molecular arms‐race’ between the nuclear and mitochondrial genomes also known as nucleocytoplasmic conflict (Touzet and Budar, 2004).

Analysis of conservation of RFL genes across the genus *Hordeum* gave striking insight into the RFL sequence retention and variability across hundreds of individual accessions and landraces, despite the fact that only about half of ‘Morex’ reference RFLs were found in the draft WGS assemblies (The International Barley Genome Sequencing Consortium, 2012) and 262 barley EC data sets (Mascher *et al*., 2013). Two main factors could have contributed to this result: (i) non‐exhaustive coverage of the sequence capture experiment; and (ii) limitations of the *de novo* assembly of RFL sequences from short reads. Based on the comparison with the barley draft genome assembly, regions covered by the capture targets were estimated to encompass ~78% of high‐confidence exonic sequence and ~41% of low‐confidence exon sequence (Mascher *et al*., 2013). Due to the repetitive nature of RFL genes, one capture probe could hybridise to several paralogous RFL regions, therefore it is rather unlikely that the EC approach is the sole cause of the low recovery of RFL sequences. Most likely, the high similarity of RFL sequences, often originating from duplications, created ambiguities in short read alignments and assemblies. This, in turn, generated shorter (partial) sequence scaffolds or chimeric sequences formed from several highly similar RFL paralogues being merged into a single sequence. These assembly issues are likely to be particularly prevalent for the larger RFL clusters containing multiple similar genes, and probably explain why, for example, the sequences missing from the draft WGS assemblies predominantly correspond to the largest cluster on chromosome 1H. Taking into account these considerations, the total number of 68 RFL POGs determined by HCA of ~7700 RFL sequences is probably an underestimate. More POGs are expected to be identified in high‐quality whole‐genome sequences obtained, ideally, in a hybrid assembly approach with long and short reads, a method that has recently been shown to improve discovery of gene family expansions in plants (Miller *et al*., 2017) and was successfully applied in the assembly of several plant genomes, including the large and highly repetitive genome of *Aegilops tauschii* (Zimin *et al*., 2017). Resolving variation in RFL gene clusters will be crucial for identifying new *Rf* gene variants and will help in understanding the evolution of this complex gene family.

For some POGs, high retention (a representative sequence present in more than ~50% of surveyed samples) across hundreds of barley accessions was observed. Four of these ‘core’ POGs show extremely high sequence conservation and, based on similarity with ‘Morex’ genes, they most likely represent singlets. On the other hand, a few of the ‘core’ RFLs show much higher nucleotide polymorphism across accessions, and their genomic locations in ‘Morex’ coincide with RFL clusters. These genes show much higher average ω values than other POGs. RFL clusters have been proposed as sites in the nuclear genome where novel *Rf* gene variants are created and selected for their ability to target novel RNAs causing plant sterility created by recombination events in the mitochondrial genome (Fujii *et al*., 2011). Previous studies have shown rapid RFL sequence divergence in interspecific comparisons (Fujii *et al*., 2011; Melonek *et al*., 2016). In this study, a much larger set of RFL sequences from closely related barley accessions and landraces was analysed, allowing for the first systematic intraspecific analyses of RFL diversity to be carried out. The large sample size means that the calculated probabilities for diversifying selection are much higher than those obtained in previous studies (Geddy and Brown, 2007; Fujii *et al*., 2011). Diversifying selection was previously detected on particular amino acid residues within PPR motifs (Fujii *et al*., 2011). The amino acid residues at positions 5 and 35, which are in direct contact with target RNAs (Shen *et al*., 2016), were reported to be under strong diversifying selection in interspecific comparisons (Fujii *et al*., 2011). In our study, residue 5 (which helps distinguish between purine and pyrimidine nucleotides; Barkan *et al*., 2012; Shen *et al*., 2016) is the major target of diversifying selection. Elevated probabilities of diversifying selection could be used as markers for detecting active *Rf* loci (i.e. those under natural selection) among the many RFL sequences that can be identified in complex genomic data sets. Rapid copy number variation of RFL sequences accompanied by equally rapid selective sequence changes contribute to the overall high sequence plasticity of the RFL family members, making it necessary to sequence every prospective restorer line – working with the reference genome alone only gives a very partial view of the diversity of RFL sequences within the gene pool.

Studies on CMS and fertility restoration in barley are still very limited and, to date, only *Rfm1* has been reported as a locus controlling fertility restoration in barley (Ui *et al*., 2015; Rizzolatti *et al*., 2017). The genomic location of the mapped *Rfm1* region in the barley ‘Morex’ reference genome does not coincide with either of the two RFL clusters on chromosome 1H and 2H, or any of the single RFL genes identified in this study. Recently, sequencing of BAC libraries developed from the barley restorer line Re08 allowed the probable identification of *Rfm1* as a PLS‐class PPR gene (Rizzolatti *et al*., 2017). So far, the only other PLS‐class *Rf* candidate was reported from sorghum (Klein *et al*., 2005). The restoring capability of these two PLS‐class genes remains to be proven and the molecular mechanism underlying the mode of action of PLS‐type *Rf* genes investigated.

As the majority of *Rf* genes identified to date in plant species belong to the RFL subclade, the RFL clusters on chromosome 1H and 2H in barley are expected to coincide with the location of genomic intervals carrying putative yet to be identified *Rf* restorer genes in *H. spontaneum,* the cytoplasm donor of *msm1* and *msm2* cytoplasms. Of particular interest is the sub‐cluster 2 on chromosome 1H, as the RFL sequences located within show high copy number and sequence variation as well as elevated probabilities for diversifying selection. It was shown in rice that several *Rf* restorer genes including *Rf1a*,* Rf1b*,* Rf4* and *Rf5* are all located within the same RFL cluster located on chromosome 10, the largest RFL cluster in the rice genome (Kazama and Toriyama, 2003; Akagi *et al*., 2004; Hu *et al*., 2012). The combination of the *H. spontaneum* mitochondrial genome sequence obtained recently (Hisano *et al*., 2016) with the RFL sequence data obtained once the *H. spontaneum* reference genome becomes available will bring new insights into the mechanisms underlying sterility and fertility restoration in barley.

Our analysis represents the most comprehensive characterisation of the PPR and RFL gene families in the genus *Hordeum*. The sequence data obtained in this study are a valuable resource that can be used in the design of sequence baits destined for capture‐based target enrichment of samples prior to next‐generation sequencing (NGS). The development of high‐throughput cost‐effective NGS‐based methods will allow screening of hundreds of elite lines and wild barley accessions, and will enable a more in‐depth analysis of sequence and structural variation of RFL family in the barley pan‐genome. The obtained sequence knowledge has the potential to accelerate genomic‐based improvement of barley elite lines and will be beneficial for the development of hybrid breeding systems based on CMS.

## **EXPERIMENTAL PROCEDURES**

### Identification of RFL sequences in genomic sequence data

The barley ‘Morex’ Refseqv1.0 genome was downloaded from the Plant Genomics and Phenomics Research Data Repository https://doi.org/10.5447/ipk/2016/34 (Mascher *et al*., 2017). The WGS assemblies of *H. pubiflorum* and *H. bulbosum* were accessed from The National Center for Biotechnology (NCBI) (https://www.ncbi.nlm.nih.gov/bioproject/) project reference number: PRJEB3404 and PRJEB3403, respectively. The PPR sequences in the genomic sequence data were identified as published recently (Cheng *et al*., 2016). Only P‐ and PLS‐class ORFs with scores above 100 and 240 (as judged by *hmmsearch* scores), respectively, were chosen for further analyses. The identification of RFL sequences was performed as described earlier (Melonek *et al*., 2016), and was based on inferring orthologous sequences using phylogenetic approaches and OrthoMCL (http://www.orthomcl.org/orthomcl/) (Li *et al*., 2003). In addition, previously identified RFL sequences from *S. bicolor* and *B. distachyon* (Fujii *et al*., 2011) and *O. sativa* indica (Melonek *et al*., 2016) were included in the study. The Circos diagrams were drawn with Circos software (Krzywinski *et al*., 2009).

### Analysis of 262 barley exome capture data sets

The barley EC data sets were downloaded from the Sequence Read Archive (SRA) (https://www.ncbi.nlm.nih.gov/sra) accession number PRJEB1810 (Mascher *et al*., 2013). The sequencing reads’ insert sizes were estimated by aligning reads with ‘Morex’ Refseqv1.0 using BWA (Li and Durbin, 2009) and subsequently were assembled with MaSuRCA v3.2.2 (Zimin *et al*., 2013). The obtained sequence scaffolds were screened for the presence of RFL genes as described above. HCA (Huang *et al*., 2010) was applied to assign the identified RFL sequences into POGs. Three iterated runs of CD‐HIT clustering with identity thresholds (‐c) of 98, 96 and 93%, respectively, were performed. The parameters of single CD‐HIT run were as follows: ‐c 0.98 ‐n 5 ‐g 1 ‐G 0 ‐aS 0.99 ‐d 0. The presence/absence matrix of POGs in the barley accessions was generated with an offline version of *plotly* (https://plot.ly/python/) in *Jupyter* notebook (http://jupyter.org/).

### Calculation of probabilities of positive/diversifying selection with CODEML

To detect positive selection, the NSites test implemented in the CODEML program from the Phylogenetic Analysis by Maximum Likelihood (PAML) package version 4.9 (Yang, 2007) was used. Neutral models were compared with alternative models allowing positive selection and performing likelihood ratio tests of the following PAML models: M1 versus M2 and M7 versus M8. For the analysis we used only RFL genes for which representatives in more than four accessions were identified. First, sequences assigned to each POG by HCA longer than 400 amino acids were aligned with Muscle (Edgar, 2004). The number of sequences included in each sequence alignment is given in Table S10. The sequence alignments were used to construct a maximum‐likelihood tree based on the JTT matrix‐based model (Jones *et al*., 1992) in MEGA software version 7.0 (Kumar *et al*., 2016). Initial trees for the heuristic search were obtained automatically by applying Neighbor‐Join and BioNJ algorithms to a matrix of pairwise distances estimated using a JTT model, and then selecting the topology with superior log likelihood value. The tree with the highest log likelihood was exported into Newick standard format that was directly used by CODEML. Sequence alignments along with the topology trees generated for each POG were deposited in the UWA Research Repository (https://doi.org/10.26182/5ba4695d7ff38) (Melonek *et al*., 2018). PAL2NAL (Suyama *et al*., 2006) was used to generate codon‐protein alignments. CODEML was run with the following settings: runmode = 0, CodonFreq = 2:F3x4, model = 0, Nsites = 0 1 2 7 8. The BEB approach implemented in CODEML (Yang *et al*., 2005) was used to identify sites potentially under positive selection.

### Sequence homology and synteny analysis

The analysis of sequence homology was performed on a set of RFLs identified earlier in sorghum and Arabidopsis (Fujii *et al*., 2011), as well as rice and *Brachypodium* (Melonek *et al*., 2016). The sequences were aligned with Muscle v.3.8.31 (Edgar, 2004) and the alignment was used to generate a tree with FastTree (Price *et al*., 2009). The tree branches were coloured in *Geneious* (http://www.geneious.com/). To study the conservation of genomic locations of RFL regions between barley and three other cereal species, a data set with chromosomal synteny reported earlier (The International Barley Genome Sequencing Consortium, 2012) was used. The figure was generated with Circos (Krzywinski *et al*., 2009).

## **Conflict of interest**

This work has been partially funded by Groupe Limagrain.

## Supporting information


**Figure S1.** Identification of RFL sequences in the genomic data sets of *H. vulgare* cvs. ‘Morex’, ‘Barke’ and ‘Bowman’.

**Figure S2.** Sequence alignment of POG15 and POG01 representative sequences.

**Table S1** Summary of RFL genes identified in the barley cv. ‘Morex’ Refseqv1.0 genome.
**Table S2** Summary of RFL genes identified in the WGS assemblies of barley cvs. ‘Morex’, ‘Barke’ and ‘Bowman’ compared with RFLs identified in the ‘Morex’ Refseqv1.0.
**Table S3** CD‐HIT clustering of ‘unanchored’ barley RFLs.
**Table S4** List of EC data sets (Mascher *et al*., 2013) used in this study.
**Table S5** Number of PPR, P‐class, PLS‐class and RFL sequences identified in the EC data sets.
**Table S6** Overlapping START and END between RFL ORFs and scaffolds.
**Table S7** Representatives of 68 POG sequences identified across 262 barley accessions.
**Table S8** Frequency of 68 POGs identified across 262 accessions.
**Table S9** ω values calculated for HvRFLs with CODEML (Model 0).
**Table S10** Comparison of codon substitution models M2 versus M1 and M8 versus M7 across POGs.
**Table S11** Sequence conservation among RFL sequences identified in *H. pubiflorum*,* H. bulbosum* and *H. vulgare*.
