# Supplementary material for: High intraspecific diversity of Restorer‐of‐fertility‐like genes in barley
Source: Plant J. 2018 Nov 9;97(2):281–95. doi: 10.1111/tpj.14115 (PMC7380019; doi:10.1111/tpj.14115)
Supplement: Supplementary file 4 [file TPJ-97-281-s004.docx]

**Supporting Information Legends**

**Figure S1.** Identification of RFL sequences in the genomic data sets of *H. vulgare* cvs. ‘Morex’, ‘Barke’ and ‘Bowman’. (**A**) P-class PPR proteins supplemented with 63 reference RFL sequences (as identified earlier (Fujii *et al.*, 2011)) were aligned with Muscle v3.8.31 (Edgar, 2004). The resulting sequence alignment was used to generate a radial tree in Geneious v8.1.6. The deeply monophyletic clade containing the RFL sequences is highlighted in red. (**B**) Phylogenetic relationships between RFL sequences identified in *H. vulgare* cvs. ‘Morex’, ‘Barke’ and ‘Bowman’. The colour coding is as follow: green indicates RFLs located on chromosome 6H, blue on chromosome 1H, orange on chromosome 2H and brown on chromosome 7H. Black colour indicates RFLs found on the unanchored chromosome (Un).

**Figure S2.** Sequence alignment of POG15 (**A**) and POG01 (**B**) representative sequences. The sequences were aligned with Muscle v3.8.31 (Edgar, 2004) and analysed in Geneious v8.1.6.

**Table S1.** Summary of RFL genes identified in the barley cv. 'Morex' Refseqv1.0 genome.

**Table S2.** Summary of RFL genes identified in the WGS assemblies of barley cvs 'Morex', 'Barke' and 'Bowman' compared to RFLs identified in the 'Morex' Refseqv1.0.

**Table S3.** CD-Hit clustering of 'unanchored' barley RFLs.

**Table S4.** List of exome capture data sets (Mascher *et al.*, 2013) used in this study.

**Table S5.** Number of PPR, P-class, PLS-class and RFL sequences identified in the exome capture data sets.

**Table S6.** Overlapping START and END between RFL ORFs and scaffolds.

**Table S7.** Representatives of 68 POG sequences identified across 262 barley accessions.

**Table S8.** Frequency of 68 POGs identified across 262 accessions.

**Table S9.** ω values calculated for HvRFLs with CODEML (Model 0).

**Table S10.** Comparison of codon substitution models M2vsM1 and M8vsM7 across POGs.

**Table S11.** Sequence conservation among RFL sequences identified in *H. pubiflorum*, *H. bulbosum* and *H. vulgare*.
